# Allorecognition in the Tasmanian Devil (*Sarcophilus harrisii*), an Endangered Marsupial Species with Limited Genetic Diversity

**DOI:** 10.1371/journal.pone.0022402

**Published:** 2011-07-21

**Authors:** Alexandre Kreiss, Yuanyuan Cheng, Frank Kimble, Barrie Wells, Shaun Donovan, Katherine Belov, Gregory M. Woods

**Affiliations:** 1 Menzies Research Institute Tasmania, University of Tasmania, Tasmania, Australia; 2 Faculty of Veterinary Science, University of Sydney, New South Wales, Australia; 3 School of Medicine, University of Tasmania, Tasmania, Australia; 4 Royal Hobart Hospital, Department of Pathology, Tasmania, Australia; La Jolla Institute of Allergy and Immunology, United States of America

## Abstract

Tasmanian devils (*Sarcophilus harrisii*) are on the verge of extinction due to a transmissible cancer, devil facial tumour disease (DFTD). This tumour is an allograft that is transmitted between individuals without immune recognition of the tumour cells. The mechanism to explain this lack of immune recognition and acceptance is not well understood. It has been hypothesized that lack of genetic diversity at the Major Histocompatibility Complex (MHC) allowed the tumour cells to grow in genetically similar hosts without evoking an immune response to alloantigens. We conducted mixed lymphocyte reactions and skin grafts to measure functional MHC diversity in the Tasmanian devil population. The limited MHC diversity was sufficient to produce measurable mixed lymphocyte reactions. There was a wide range of responses, from low or no reaction to relatively strong responses. The highest responses occurred when lymphocytes from devils from the east of Tasmania were mixed with lymphocytes from devils from the west of Tasmania. All of the five successful skin allografts were rejected within 14 days after surgery, even though little or no MHC I and II mismatches were found. Extensive T-cell infiltration characterised the immune rejection. We conclude that Tasmanian devils are capable of allogeneic rejection. Consequently, a lack of functional allorecognition mechanisms in the devil population does not explain the transmission of a contagious cancer.

## Introduction

Devil facial tumour disease (DFTD) is a transmissible Schwann cell cancer of Tasmanian devils (*Sarcophilus harrisii*) [1], [2], [3]. The cancer cells are passed from animal to animal during the process of biting, which most frequently occurs on the head and neck. Metastases to distant organs are common and affected devils die within months after tumours become apparent [2], [4]. This disease was noted in 1996 in the far northeast of Tasmania, and has since spread to over half of the devil's range [5], [6]. The far northwestern areas of Tasmania still remain disease free, although it is predicted that DFTD will affect all devil habitat within the decade. It is possible that extinction of the species will occur within 20 years [5].

Despite being a cell allograft, DFTD tumours do not evoke an immune response [7], and there is no evidence of lymphocyte infiltration into the tumour masses [8]. In vertebrates, organ, tissue or cell transplants are rejected by the immune system, unless the recipient is severely immunocompromised or both recipient and donor share identical Major Histocompatibility Complex (MHC) genes (e.g. homozygous twins) [9]. MHC genes were originally identified in mammalian cells, encoding specialised glycoproteins expressed on the cell surface of almost all nucleated cells. These genes were first related to foreign tissue (allograft) transplantation, but are now known to be essential in the immune recognition of pathogens and tumour cells [10], [11]. There are two main MHC subgroups that have different immunological functions, MHC I and MHC II. MHC I molecules are composed of a polymorphic α-chain associated with a non-polymorphic β2-microglobulin. Its role is to present intracellular antigens to CD8+ T-cells. These molecules are present on all nucleated cells. MHC II molecules are composed of a polymorphic α-chain attached to a polymorphic β-chain, and present extracellular antigens to CD4+ T-cells. Under normal conditions, MHC II is only located on the cell surface of professional antigen presenting cells, such as dendritic cells and macrophages (reviewed in [12], [13]). Tasmanian devils have a competent immune system [14], [15], but the devil population has been through genetic bottlenecks and lacks MHC diversity [16], [17].

MHC molecules regulate the immunological mechanisms of tissue graft rejection and provoke vigorous T-cell responses against incompatible cells [18], [19]. Different effector processes may reject the allografts, but the main mediators are CD8+ T-cells and CD4+ T-cells. Allopeptides presented to CD8+ T-cells by MHC I molecules stimulate the differentiation of cytotoxic T-cells, which kill nucleated cells expressing MHC I in the graft. Alloantigens presented by MHC II molecules activate helper T-cells, which differentiate and produce cytokines that damage the tissue graft. Allograft rejection mediated by CD4+ and CD8+ T cells is deemed acute rejection, as it can usually occur within eight to 12 days after the transplant. This form of rejection is primarily due to mismatching of MHC loci [20], [21].

A predictive *in vitro* test for T-cell recognition of allogeneic MHC molecules is the mixed lymphocyte reaction (MLR) [22]. This experiment has been used in clinical analyses of allogeneic impact of MHC I and II mismatches between live donors (e.g. kidney donors) and recipients. Donors with poor MLR responses against the recipient have lower incidence of graft rejection [23], [24]. MLR experiments can be performed with cultured lymphocytes from both donor and recipient and the resultant proliferation will correspond to differences in the MHC alleles of both individuals (two-way MLR). By inactivating the mononuclear cells from the donor (by X-irradiation or chemical mitotic inhibition), only the recipient response is evaluated (one-way MLR).

Skin grafting between unrelated individuals has been performed to test MHC disparity in a wild mammal, the cheetah (*Acynonyx jubatus*). Captive cheetahs were unable to quickly recognize alloantigens as foreign and reject the skin grafts [25]. More recently, wild-living Namibian cheetahs were shown to have low levels of MHC variation, although this did not appear to impair their immune response against infectious diseases [26], [27]. To date, a lack of MHC diversity has been the primary reason given for the lack of rejection of allograft tumours by Tasmanian devils. It has been proposed that devils have a similar range of MHC antigens as the tumours [16], [17], and therefore do not see the tumours as foreign. Acceptance, or delayed rejection, of skin allografts would help to confirm the theory that impoverished MHC diversity is responsible for a lack of tumour allograft recognition [28], [29]. To determine whether this limited MHC diversity is sufficiently low to permit allograft transplantation, we characterised levels of functional MHC variation by two-way MLR and skin grafts experiments.

## Materials and Methods

### Ethics statement

All experiments describing the use of animals were undertaken with approval and under inspection of the Animal Ethics Committee of University of Tasmania, permit numbers A9491 and A11052. Captive devils were housed in groups of two or three in 100 m^2^ enclosures. Animals were fed possum or wallaby meat once a day and water *ad libitum*. All procedures (blood collection, skin graft surgery, biopsy and bandage removal) were performed with the animals under general anaesthesia and all efforts were made to minimise pain or discomfort.

### Two-way mixed lymphocyte reaction

**Figure 1:**
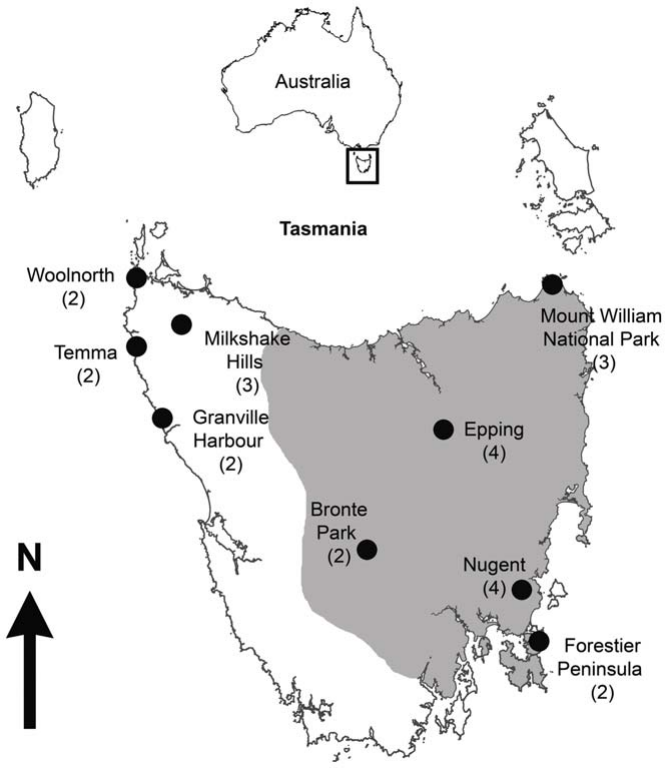
Origin of Tasmanian devils used for mixed lymphocyte reactions and skin graft surgery. Mount William National Park, Epping, Bronte Park, Nugent and Forestier Peninsula were considered eastern sites, whereas Woolnorth, Temma, Milkshake Hills and Granville Harbour were considered western sites. The grey area illustrates the DFTD-affected area in 2010 (http://tinyurl.com/3b6d8qf). Number of devils used in MLR experiments from each area is shown in brackets.

Fifteen Tasmanian devils from five areas in eastern Tasmania (Nugent, Epping, Mount William National Park, Forestier Peninsula and Bronte Park) and nine devils from four regions in western Tasmania (Woolnorth, Temma, Granville Harbour and Milkshake Hills) were used. Figure 1 shows a map of Tasmania with the geographical locations of all devils used for MLR and skin graft experiments. The DFTD-affected area is also illustrated. Whole blood was collected into lithium heparin tubes from the jugular vein while under general anaesthesia. Mononuclear cells were harvested using density gradient centrifugation as described previously [14]. Cells were diluted to a concentration of 10^6^ cells/mL in RPMI 1640 incomplete medium (CSL Limited 05182301) supplemented with 10% pooled devil plasma, 2 mM of glutamine (Sigma G7513) and 15 mg of gentamicin (Pfizer 61022010).

MLR experiments were conducted in U-bottomed 96 well plates (Iwaki 3870-096), mixing 100 µL of a mononuclear cell suspension from one devil with 100 µL of a mononuclear cell suspension from another devil. Background controls consisted of 200 µL of mononuclear cells from individual devils incubated alone and positive controls consisted of mononuclear cells of individual devils incubated with 50 µg/mL of Concanavalin A (Con A, Sigma C7275). Mixed lymphocyte cultures were incubated at 37°C and 5% CO_2_ for 144 hours. Mitogen controls were incubated under the same conditions for 96 hours. Eighteen hours prior the harvesting, cultures were pulsed with 1 µCi tritiated thymidine (Amersham Pharmacia Biotech TRA 310) to assess proliferation. Cells were harvested onto a filter paper and dried overnight at room temperature. Uptake of thymidine by proliferating cells was measured by radiation emittance using a scintillation counter (Pharmacia, 1214 Rackbeta). Results were obtained in counts per minute (CPM) and stimulation indices (SI) were calculated with the formula: SI = Average CPM of mixed culture/Average CPM of control cultures. Mitogen stimulated culture controls were calculated by dividing the average of incorporated thymidine in stimulated cultures by non-stimulated cultures. All SI controls were considered to be one in the MLR experiments, as no proliferation was characterised in autologous cultures. All experiments were conducted in triplicates. MLR results were considered strong when SI >10, moderate when 5<SI<10 and low or no reaction when SI<5. To compare differences in MLR stimulation indices between groups and between ‘eastern versus eastern’, ‘western versus western’ and ‘eastern versus western’, results were log transformed and analysed with nonparametric Mann Whitney test with non-Gaussian distribution.

### Skin graft procedures

Seven unrelated captive devils of eastern Tasmania were used for two-way skin grafting experiments (one of the animals was used for two procedures). All devils were three year old females, except TD 187, which was a three year old male. Table 1 shows the pairing and origin of devils for each procedure. Surgeries were performed in two devils simultaneously. Animals were pre-medicated with acepromazine (0.2 mg/Kg) and morphine (1 mg/Kg) injected subcutaneously. For further analgesia, xylocaine 1% was injected in the subcutaneous tissue around the surgery site (not exceeding 20 mg/Kg). General anaesthesia was induced by isofluorane delivered via an endotracheal tube. The hair of the surgery site (the dorsal area of the animal) was clipped and shaved and the site disinfected with a solution of chlorexidine and cetrimide. The surgery field was covered with a sterile fenestrated drape.

**Table 1:** Origin and pairing of Tasmanian devils used for skin graft experiments.

Tasmanian devil ID	Origin
TD 190 and TD 199	Nugent and Epping
TD 187 and TD 200	Nugent and Mount William National Park
TD 188 and TD 189	Forestier Peninsula and Nugent
TD 190 and TD 191	Nugent and Forestier Peninsula

A square (3×8 cm) was drawn on the skin of the mid-dorsal region of each devil and a dermatome knife with a sterile blade was used to ‘shave’ 1 mm thick dermis and epidermis of the demarcated skin. This loose piece of skin was divided into approximately two equal pieces (approximately 3×4 cm) and one of them slid in to the cranial wound (autograft). The other piece (allograft) was placed in sterile Hartmann's solution, until the same procedure had been performed on the other devil. The allograft skin pieces were then placed in the caudal position of the wound. Surgical glue was placed on the borders of the grafts to secure the skin. A sterile dressing was applied to the surgical wound and the thoracic and chest areas were covered with cotton wool and elastic bandages under firm pressure. Bandages were changed on Day 7 and removed on Day 14. Pain relief medication (meloxicam 0.05 mg/Kg) was administered in the food for three to five days after skin graft surgery.

### Monitoring of the grafts

On Days 7, 14 and 21 the grafts were visually inspected for signs of rejection. The grafts were photographed and a punch biopsy (3–4 mm) was taken from the grafts and fixed in 10% buffered formalin solution for one to four weeks. After fixation, skin biopsy punches were processed and paraffin embedded. Four to six sections (3 µm thickness) were cut onto 3-aminotriethoxysilane (Sigma A7222) coated slides for haematoxylin and eosin staining and the same number of sections was prepared for immunohistochemistry.

### Immunohistochemistry

Skin biopsies were labelled for T-cells with a rabbit anti-human CD3 antibody (Dako A0452). Isotype controls included non-specific rabbit immunoglobulin fraction (Dako X0903) and negative controls were labelled without the primary antibody (replaced with antibody diluent, Dako S0809). Sections of devil lymph nodes were used as positive controls.

Tissue sections were deparaffinised in xylene and rehydrated through graded alcohol solutions to water and boiled in citrate buffer solution (pH 6) in an electric pressure cooker for 10 minutes at medium heat. Slides were left to cool to 35°C and placed in PBS. Endogenous peroxidase activity was quenched by incubating sections with a solution of 3% H_2_O_2_ for 15 minutes and non-specific protein binding was blocked with serum-free protein block solution (Dako X0909) for 30 minutes. The antibody was diluted (1∶800) with antibody diluent and placed onto the slides for 60 minutes. Antibody binding was detected by placing a biotinylated link universal followed by streptavidin and horseradish peroxidase (30 minutes each) (LSAB kit, Dako K0690). A solution of diaminobenzidine (Dako K3466) was placed onto the slides for ten minutes to allow for brown colour development of positive cells and then washed with distilled water. Sections were briefly counterstained with haematoxylin for 40 seconds, dehydrated through graded alcohol solutions to xylene and coverslipped.

Histology and CD3-labelled sections were examined for signs of immunological rejection and a pathological score adapted from a previous study in human hand transplantation [30] was given to each section. Table S1 describes the criteria used to determine immune rejection.

### Genotyping of skin graft recipients and donors

The seven individuals used for skin graft experiments were genotyped at MHC I α chain and MHC II β chain loci through nucleotide sequencing. Genomic DNA samples were extracted from fresh or frozen whole blood using MoBio UltraClean BloodSpin Kit. The α1 domain of MHC I genes and β1 domain of MHC II genes were amplified by PCR using previously published primers and PCR conditions [17], [31]. PCR amplifications were performed on a Bio-Rad MJ Mini Personal Thermal Cycler and the Platinum *Taq* DNA Polymerase High Fidelity Kit (Invitrogen 11304-011) was used to ensure the lowest error rate. PCR products were isolated by running 1.8% agarose TBE gels using Bioline HyperLadder IV as size marker and purified from the gel using UltraClean 15 DNA Purification Kit (MoBio 12100-300). Purified DNA fragments were cloned in a pGEM-T Easy Vector (Promega A1360) /JM109 High Efficiency Competent Cells (Promega L1001) cloning system. Twenty-four clones were picked for each sample. Plasmids were extracted using UltraClean 6 Minute Mini Plasmid Prep Kit (MoBio 12300-250) and sequenced in two directions with T7 and SP6 primers at the Australian Genome Research Facility. Two independent PCRs were performed for each individual, and only sequence variants found in more than one PCR amplification were included in the subsequent analyses to minimize nucleotide errors yielded during PCR, cloning and sequencing. The sequencing results were quality-checked in Sequencher 4.1.4 (Gene Codes) and aligned with previously identified devil MHC alleles [17] in BioEdit using the ClustalW alignment tool [32], [33].

## Results

### Tasmanian devils respond in mixed lymphocyte reactions

**Figure 2:**
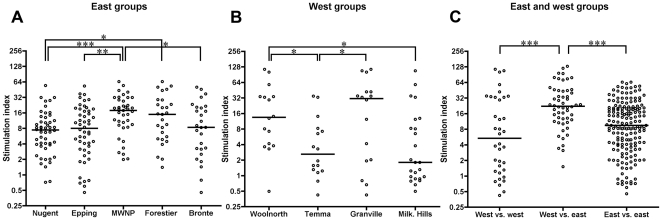
Mixed lymphocyte reaction between groups. Results are presented as stimulation indices and median is shown. Statistical significance is illustrated as *p<0.05, **p<0.005 and ***p<0.0001. A. Mixed lymphocyte reactions within eastern groups. Devils from Mount William National Park had the highest median proliferations, followed by devils from Forestier Peninsula. B. Mixed lymphocyte reactions within western groups. Devils from Granville Harbour had the highest stimulation indices compared to the other groups. C. Mixed lymphocyte reactions between eastern and western groups. Median stimulation indices were highest when experiments were conducted between eastern and western devils.

**Figure 3:**
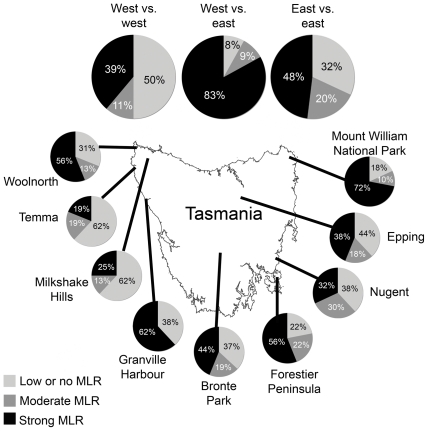
Intensity of MLR responses among all groups. Intensity of MLR responses varied greatly among devils. The greatest responses occurred in MLR between devils from east and west Tasmania.

To confirm that the mononuclear cell suspensions were able to proliferate, all suspensions were incubated with the mitogen Con A. Each of these controls resulted in high SI, indicating that experiment conditions were optimum (data not shown). There was a wide range of MLR responses among all groups, from SI<1 to SI>100. Figure 2a summarises the MLR proliferations among the eastern groups. The MLRs from devils from Mount William National Park showed the highest SI, whereas the MLRs from devils from Nugent had the lowest responses. Figure 2b illustrates the MLR responses among the western groups. The MLRs from devils from Granville Harbour and Woolnorth had the highest proliferations, whereas MLRs from devils from Milkshake Hills and Temma had the lowest responses. Because DFTD is spreading from eastern to western areas of Tasmania, we investigated the functional MHC diversity between devils from these two areas. Figure 2c shows that the MLR responses from west versus east devils were significantly higher than the MLR responses from east versus east devils and west versus west devils. In order to compare these results more effectively, the SI was characterised as either strong (SI>10), moderate (5<SI<10) or low or no reaction (SI<5). Most MLRs between devils from east and west yielded strong responses. Figure 3 illustrates the intensity of MLR responses among all groups.

### Tasmanian devils reject skin allografts

Skin graft surgeries were performed to test whether allogeneic tissue from an unrelated devil would be immunologically accepted by the recipient devil. Five of the eight skin allografts and six of the eight autografts engrafted successfully, as determined by macroscopic and histological assessment. The remaining three allografts and two autografts showed pathological changes characteristic of mechanical trauma, resulting in failure of these tissues to engraft. The main histological alterations in these unsuccessful grafts were necrosis associated with polymorphonuclear cell infiltration, surface parakeratosis and fibrin deposition.

**Figure 4:**
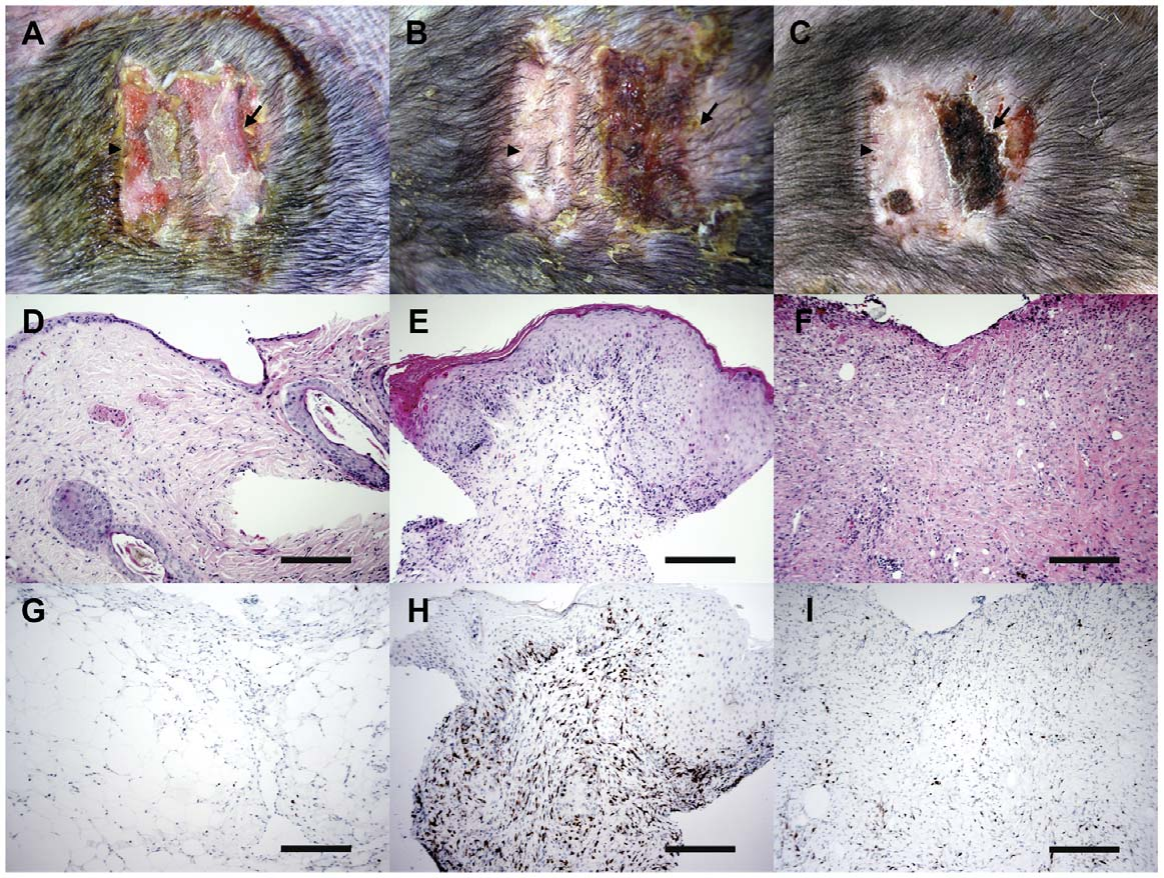
Skin graft between unrelated Tasmanian devils. A–C: Macroscopic appearance of the autograft (arrowhead) and allograft (arrow), at seven, 14 and 21 days after surgery. Dark dots in the grafts are scars from punch biopsies. D–F: Histological appearance of allografts at seven, 14 and 21 days after surgery, respectively. Note extensive mononuclear infiltration at 14 days after the surgery (E), and loss of epidermis at 21 days after surgery (F). Scale bars represent 200 µm. G–I: CD3 labelling of allografts at seven, 14 and 21 days after surgery, respectively. Note the extensive CD3 infiltration at 14 days after surgery (H). Scale bars represent 200 µm.

All successful grafts appeared indistinguishable from the autografts at Day 7. Four of five engrafted allografts had macroscopic changes associated with immune rejection 14 days following the surgery. These changes consisted of scaly lesions, coagulative exudates and brown to black coloration of the allografted skin. For one allograft, these macroscopic changes were not visible until 17 days after the procedure. Microscopically, immune rejection was characterised as Grade II, III or IV rejection (moderate, severe or very severe, respectively). Typical alterations were moderate to severe perivascular and interstitial CD3 infiltration, dermal and epidermal lymphocytic exocytosis, usually accompanied by apoptotic keratinocytes and epidermal necrosis. Spongiosis was a common finding. Most of these alterations were recognised 14 days after the surgery, and progressed to very severe rejection on Day 21. In three cases, there was total loss of the allografted skin, hence a biopsy could not be taken. Table 2 summarises the results for all surgeries (Table S2 describes the pathological changes of the allografts in detail) and Figure 4 shows the macroscopic and microscopic appearance of a representative result from one experiment.

**Table 2:** Outcome of skin allografts.

Tasmanian devil ID	Day 7	Day 14	Day 21
TD 190	Grade II	Grade IV	Necrotic skin, biopsy not done
TD 199	No evidence of rejection	Grade III	Grade IV
TD 187	Unable to determine rejection	Grade IV	Necrotic skin, biopsy not done
TD 188	No evidence of rejection	Grade III	Grade IV
TD 191	No evidence of rejection	Grade III	Necrotic skin, biopsy not done

TD 190 was the only devil that showed an early rejection response at Day 7. All five devils had Grade III to Grade IV rejection at Day 14 and Day 21.

### MHC I and II genotyping

MHC genotyping and MLR results for animals used for the skin graft surgeries are summarized in Table 3 and Table 4. TD 190 and TD 199 have exactly the same set of MHC I and II alleles. TD 188 and TD 189 have three (two MHC I and one MHC II) allelic mismatches, resulting in amino acid variations at two peptide binding sites in MHC I α1 domain. The other two pairs of individuals both have four MHC allele mismatches but no different amino acid substitutions at peptide binding sites were identified.

**Table 3:** MHC genotyping of Tasmanian devils used for skin graft experiments.

Tasmanian devil ID	MHC I α1 sequence variants	MHC II β1 sequence variants
TD 190	SahaI*27, 28, 32, 35, 49	SahaDAB*01, 03, 05
TD 199	SahaI*27, 28, 32, 35, 49	SahaDAB*01, 03, 05
TD 187	SahaI*28, 32, 35, 49, 57	SahaDAB*01, 03, 05, 12
TD 200	SahaI*27, 28, 32, 35, 49	SahaDAB*01, 03, 05, 11
TD 188	SahaI*27, 32, 35, 49	SahaDAB*01, 03, 05, 15
TD 189	SahaI*27, 32, 35, 48	SahaDAB*01, 03, 05, 11, 15
TD 191	SahaI*28, 32, 34, 48, 49	SahaDAB*01, 03, 05, 13

TD 190 and TD 199 shared all MHC I and II alleles. The remaining devil pairs had two to three MHC I allelic mismatches and one to two MHC II allelic mismatches.

**Table 4:** Amino acid difference count at peptide binding sites and MLR results within skin graft devil pairs.

Tasmanian devil ID	Amino acid difference count at peptide binding sites at MHC I α1	Amino acid difference count at peptide binding sites at MHC II β1	Mixed lymphocyte reaction (SI)
TD 190 and TD 199	0	0	1
TD 187 and TD 200	0	0	1
TD 188 and TD 189	2	0	17
TD 190 and TD 191	0	0	5

TD 188 and TD 189 had two amino acid differences at peptide binding sites at MHC I α1 and had a strong MLR response. The other three pairs did not have amino acid difference count at peptide binding sites at MHC I α1 or MHC II β1 and had low MLR responses.

## Discussion

The presence of a histocompatibility system should prevent the establishment of transmissible cancers in vertebrates [34], [35]. Despite this, in addition to DFTD, two other naturally transmissible tumours in mammals exist. Canine transmissible venereal tumour (CTVT) [36], which affects members of the Canidae family. A transmissible sarcoma in captive Syrian hamsters (*Mesocrycetus auratus*), which was either artificially or spontaneously transmitted by social interactions [37]. CTVT down-regulates MHC antigens [38] and secretes immunosuppressive molecules [39] to escape the immune system. Syrian hamsters bearing transmissible sarcomas originated from a single family [40], suggesting that they were highly monomorphic at the MHC loci. The mechanism of DFTD tumour acceptance is still unclear. Low MHC variation between devils is the most accepted hypothesis to explain the susceptibility of the species to this cancer [16].

Even though Tasmanian devils lack MHC diversity [17], it is becoming apparent that devils are capable of allogeneic responses. Our previous work showed low MLR responses using pooled lymphocytes as stimulators [16]. It is likely that different (or rare) MHC antigens were ‘diluted out’ and not present in enough quantity to stimulate proliferation of the responder cells. The present experiments produced responses throughout the devil range, although some MLR experiments yielded low or no proliferations, suggesting that these devils share most MHC antigens. Importantly, some high responses were identified in the MHC-impoverished eastern population, in which DFTD has spread rapidly. The greatest MLR responses were found between eastern and western devils, which possess differences in the MHC nucleotide sequence [17]. We confirmed this by sequencing MHC I and II alleles from two western devils (data not shown). These animals had three MHC I and two MHC II alleles that were not present in the eastern population analysed. This indicates that allelic and antigenic differences might be behind the higher allogeneicity between the two populations. The MLR results suggest that unaltered allogeneic cells should initiate an immune response in most recipient devils, irrespective of the origin of the animal.

All successful allografts were acutely rejected, despite low MLR responses in three out of the four skin graft pairs and little or no mismatches in MHC I and II alleles. The rejection of the foreign tissue was usually visible macroscopically and microscopically by 14 days after the procedure. The grafts could only be assessed every seven days, as procedures with live devils require general anaesthesia. In this context, the onset of rejection occurred between seven and 14 days, which is within the normal range (8–12 days) [41], [42]. The low MLR responses and the combined MHC I and II genotyping would suggest that a slower rejection should have occurred. This was not the case, suggesting that little mismatches can activate an allogeneic response in devils. Indeed, allografts in animal models may only require a single amino acid difference between donor and recipient to be rejected [43], [44]. In addition, other genetic (such as cytokine genes and killer cell immunoglobulin-like receptor haplotypes) and non-genetic (such as age, source of skin and size of the graft) factors may also affect the transplant outcome [45], [46], [47]. In human transplantation, allografts from fully matched unrelated donors can still be rejected, unless the recipient receives immunosuppressive therapy [48], [49]. The advantage of using skin transplants is that the outcome of the experiments is easily visualised. The disadvantage is that skin is a very immunogenic tissue and might be rejected more rapidly than other organs [42], [50]. Skin harbours cells expressing MHC I and II, plus minor histocompatibility complex molecules, all of which can act as alloantigens and trigger rejection. Nonetheless, it is clear that little antigenic or allelic mismatches are sufficient to trigger rapid rejection of foreign tissue between unrelated devils.

The florid T-cell infiltration observed in the skin allografts contrasts with the absence of tumour infiltrating lymphocytes in DFTD tumours [4], [8]. Down-regulation of the MHC machinery is a well-known mechanism that tumour cells, including CTVT, utilise to escape destruction by T-cells [38], [51], [52], [53]. The expression of MHC I and II proteins by DFTD tumour cells still needs to be resolved. DFTD tumour cells express MHC I and II at mRNA level [16], but it is unknown whether this is translated into functional proteins. Preliminary experiments in our laboratory indicate that DFTD tumour cells do not express MHC II on the cell surface (unpublished data). It has not been possible to test for MHC I expression, due to the lack of specific antibodies. In tumours with changed MHC profile, NK cells are activated and promote the killing of abnormal cells [54]. Although devils possess the expected range of immune cells [55], NK cell function has still to be explored in the Tasmanian devil. Tumours can utilise other mechanisms to evade immune recognition. Increased resistance to cytotoxic molecules, such as perforin [56], decreased tumour antigen expression [57] and secretion of immunosuppressive factors, such as IL-4, IL-10 and transforming growth factor-β1 [58], [59] are alternative (or concurrent) mechanisms of immune escape.

The devil population is under threat because an allogeneic tumour cell clone is being transferred between animals without immune recognition. The host is immunocompetent [14], [15], but the population has undergone bottlenecks in the past and lacks genetic and MHC diversity [16], [17], [60]. This homogeneity in the population, however, is not sufficient to allow allotransplantation between unrelated devils. DFTD tumour cells are likely to have evolved adaptive mechanisms to survive unhindered in the tissues of the new host.

## Supporting Information

Table S1 **Skin graft scoring used for the Tasmanian devil skin grafts.**
(DOC)

Table S2 **Histopathological alterations of skin allografts of Tasmanian devils.**
(DOC)
