# Supplementary material for: Allorecognition in the Tasmanian Devil (Sarcophilus harrisii), an Endangered Marsupial Species with Limited Genetic Diversity
Source: PLoS One. 2011 Jul 21;6(7):e22402. doi: 10.1371/journal.pone.0022402 (PMC3141043; doi:10.1371/journal.pone.0022402)
Supplement: Table S1 — Skin graft scoring used for the Tasmanian devil skin grafts. (DOC) [file pone.0022402.s001.doc]

**Table S1. Skin graft scoring used for the Tasmanian devil skin grafts.**

| Grade | Macroscopic appearance | Microscopic changes |
| --- | --- | --- |
| Grade 0: no rejection | Normal skin | No histological changes or occasional small number of lymphocytes near blood vessels. |
| Grade I: mild rejection | Normal skin | Mild lymphocytic infiltration present. Epidermis unaffected. Sparse CD3+ cells in the dermis. |
| Grade II: moderate rejection | Normal skin | Mildly dense infiltrate, predominantly lymphocytic, in the dermis, with perivascular cuffs spreading between collagen bundles. Occasional histiocytes or epithelioid cells between collagen bundles. Epidermis is either not affected or shows some exocytosis and/or spongiosis. Presence of sparse to moderate CD3+ cell infiltration in the dermis. |
| Grade III: severe rejection | Skin with brown colouration, exudates and scaly papules. | Evident dense lymphocytic infiltration (mainly CD3+) around blood vessels of the dermis and epidermis. Epidermis may present with spongiosis or exocytosis and some apoptotic keratinocytes. Extensive to severe CD3 infiltration in the dermis and possibly in the epidermis. |
| Grade IV: very severe rejection | Necrotic, dry with a marked brown to black colouration of the skin. A tendency for the biopsy to lose attachment to the adjacent epidermis and underlying dermis. | Confluent epidermal necrosis associated with epidermal lymphocytic exocytosis and lymphocytic infiltration of the dermis. Extensive to severe CD3+ cell infiltration. |

Adapted from [30].
