# Supplementary material for: Allorecognition in the Tasmanian Devil (Sarcophilus harrisii), an Endangered Marsupial Species with Limited Genetic Diversity
Source: PLoS One. 2011 Jul 21;6(7):e22402. doi: 10.1371/journal.pone.0022402 (PMC3141043; doi:10.1371/journal.pone.0022402)
Supplement: Table S2 — Histopathological alterations of skin allografts of Tasmanian devils. (DOC) [file pone.0022402.s002.doc]

**Table S2. Histopathological alterations of skin allografts of Tasmanian devils.**

| Day | TD 190 |
| --- | --- |
| Day 7 | Grade II (moderate) rejection. A perivascular and interstitial, moderate predominantly lymphocytic inflammatory cell infiltrate was noted within the upper half of the dermis. The overlying epidermis showed mild spongiosis with scattered lymphocytic exocytosis. No necrotic keratinocytes were seen. Extensive CD3+ cells infiltrating the dermis. |
| Day 14 | Grade IV (very severe) rejection. Confluent necrosis of the full thickness of the squamous epithelium. No viable epithelium was noted. Moderate infiltrate of CD3+ cells, few in the epidermis. |
| Day 21 | Loss of skin, biopsy not done. |
| Day | TD 199 |
| Day 7 | No evidence of rejection. Sparse to moderate CD3+ cell infiltration in the dermis. |
| Day 14 | Grade III (severe) rejection. A mild to moderate, perivascular and interstitial, predominantly lymphocytic inflammatory cell infiltrate was seen within the upper dermis associated with epidermal spongiosis and scattered, individual necrotic keratinocytes. Severe CD3+ cell infiltrate in the dermis and epidermis. |
| Day 21 | Grade IV (very severe) rejection. Confluent necrosis of the full thickness of the squamous epithelium was seen. No viable epidermis was noted. Moderate CD3+ cell infiltration. |
| Day | TD 187 |
| Day 7 | Surface parakeratosis with fibrin deposition and florid neutrophil infiltration and epidermal spongiosis. A significant dermal lymphocyte infiltrate is not identified under H&E staining. Moderate to extensive CD3+ cell infiltrate in the dermis with few infiltrating the epidermis. Unable to differentiate between mechanical trauma and immune rejection. |
| Day 14 | Grade IV (very severe) rejection. Full thickness confluent necrosis with surface parakeratosis. Significant lymphocytic infiltration in the dermis. Extensive to severe CD3+ cell infiltrate in the dermis and epidermis. |
| Day 21 | Loss of skin, biopsy not done. |
| Day | TD 200 |
| Day 7 | Focal confluent necrosis. Intact squamous epithelium showed overlaying parakeratosis, fibrin and neutrophil collections. A significant dermal lymphoid infiltrate is not seen. Sparse CD3+ cell infiltrate in the dermis. Mechanical trauma is favoured over immune rejection. |
| Day 14 | These sections had similar alterations to Day 7, with sparse to moderate CD3+ cell infiltrate in the dermis. Mechanical trauma is favoured over immune rejection. |
| Day 21 | Loss of skin, biopsy not done. |
| Day | TD 188 |
| Day 7 | Sparse CD3+ cells in the dermis. No other abnormalities observed. |
| Day 14 | Grade III (severe) rejection. Dermal lymphocytic, perivascular and interstitial infiltrate associated with prominent necrotic keratinocytes and lymphocytic exocytosis. Confluent epidermal necrosis was not seen. Extensive to severe CD3+ cell infiltrate in the dermis. |
| Day 21 | Grade IV (very severe) rejection. Confluent epidermal necrosis. Extensive CD3+ cell infiltrate in the dermis. |
| Day | TD 189 |
| Day 7 | Sparse CD3+ cell infiltrate in the dermis. No evidence of rejection. |
| Day 14 | Parakeratosis with spongiosis and focal neutrophil collections. Moderate CD3+ cell infiltration in the dermis. Favour mechanical trauma. |
| Day 21 | Confluent epidermal necrosis with neutrophil rich inflammatory cell infiltrate. Sparse CD3+ cell infiltrate in the dermis. These alterations indicate mechanical trauma. |
| Day | TD 190 (second surgery) |
| Day 7 | Parakeratosis and neutrophil collections. No significant CD3+ cells in the dermis. Consistent with mechanical trauma. |
| Day 14 | Parakeratosis and few neutrophils infiltrating the dermis. Sparse CD3+ cells in the dermis. Mechanical trauma is favoured over immune rejection. |
| Day 21 | Loss of skin, biopsy not done. |
| Day | TD 191 |
| Day 7 | Sparse CD3+ cell infiltrate in the dermis. No other abnormalities observed. |
| Day 14 | Grade III (severe) rejection. Surface parakeratosis with neutrophil rich inflammatory cell infiltrate, but intense CD3+ cells in the dermis, indicating immune rejection. |
| Day 21 | Loss of skin, biopsy not done. |
